# Morphological analysis of Apolipoprotein E binding to Aβ Amyloid using a combination of Surface Plasmon Resonance, Immunogold Labeling and Scanning Electron Microscopy

**DOI:** 10.1186/s12896-019-0589-4

**Published:** 2019-12-11

**Authors:** Tohidul Islam, Anna L. Gharibyan, Cheng Choo Lee, Anders Olofsson

**Affiliations:** 1 Department of Medical Biochemistry and Biophysics, Umeå University, SE-901 87 Umeå, Sweden; 2 Umeå Core Facility for Electron Microscopy (UCEM), Umeå University, SE-90187 Umeå, Sweden

**Keywords:** Aβ, ApoE, Immunogold, Surface plasmon resonance, SPR, Scanning electron microscopy, SEM, Fibrils, Morphology, Abeta

## Abstract

**Background:**

Immunogold labeling in combination with transmission electron microscopy analysis is a technique frequently used to correlate high-resolution morphology studies with detailed information regarding localization of specific antigens. Although powerful, the methodology has limitations and it is frequently difficult to acquire a stringent system where unspecific low-affinity interactions are removed prior to analysis.

**Results:**

We here describe a combinatorial strategy where surface plasmon resonance and immunogold labeling are used followed by a direct analysis of the sensor-chip surface by scanning electron microscopy. Using this approach, we have probed the interaction between amyloid-β fibrils, associated to Alzheimer’s disease, and apolipoprotein E, a well-known ligand frequently found co-deposited to the fibrillar form of Aβ in vivo. The results display a lateral binding of ApoE along the amyloid fibrils and illustrates how the gold-beads represent a good reporter of the binding.

**Conclusions:**

This approach exposes a technique with generic features which enables both a quantitative and a morphological evaluation of a ligand-receptor based system. The methodology mediates an advantage compared to traditional immunogold labeling since all washing steps can be monitored and where a high stringency can be maintained throughout the experiment.

## Background

Fibrillar aggregates of the amyloid β peptide (Aβ) are considered as one of the hallmarks of AD and their ultrastructural morphology and properties have been extensively studied. The Aβ-fibrils are represented by a β-sheet polymer where the lateral assembly of several thinner filaments constitute the final fibrillar morphology [1–3]. The formation of Aβ amyloid fibrils follows a nucleation-dependent path of aggregation. Here an initially formed assembly of peptides acts as a template (a nucleus) for the subsequent incorporation of monomers resulting in a highly ordered fibrillar morphology of indefinite length. Similar to the growth of a crystal, a repeating structure is propagated. Although a single fibril represents a highly ordered structure a morphological heterogeneity is frequently observed and several fibrillar forms can usually be identified within the same sample. Understanding the mechanistic details of Aβ assemblies is of interest in the design of therapeutic interventions [4, 5]. In vivo, the intrinsic properties of the Aβ peptide to form an amyloid are suppressed by several factors including degradation by neprilysin [6] and insulin-degrading enzyme [7]. A few endogenous proteins have also been found to interfere with the process of Aβ amyloid formation, including proteins containing a BRICHOS domain [8–10], transthyretin (TTR) [11–15], clusterin [16], and ApoE [17–20]. ApoE is in this context of specific interest where the ε4 allele today represents the strongest genetic linker to the development of late onset AD [21]. Identification of the mechanism of binding including a morphological evaluation of the binding pattern is therefore of interest.

Immunogold labeling in combination with electron microscopy represents a technique used for the ultra-structural investigation of biological samples. The methodology reports the binding of e.g. an antibody to its antigen or a ligand to its receptor by conjugation to a gold particle and hence facilitates detailed structural information of the ultrastructural morphology. Immunogold techniques are however frequently hampered by the ability to remove unspecific low-affinity binding and hence to obtain a high stringency of the system. Consequently, it is often difficult to discriminate between relevant binding and non-specific interactions.

Within the SPR technique, the sample for analysis is frequently attached to a surface via strong covalent bonds and the immobilized proteins can then be probed by potential interaction partners through injections into a continuous flow of the media. The methodology facilitates detailed monitoring of binding kinetics including K_D_ determination [22]. It is hence easy to monitor the removal of unspecific low-affinity interactions and the stringency of the system.

An SPR experiment frequently involves the acquired kinetic data from an interaction between e.g. a receptor and its ligand and in most cases this covers the required needs. The SPR technique has however been extensively used regarding the monitoring of amyloid formation [23–26] where a fibrillar ultrastructure is formed. Since an SPR chip is coated with a gold-surface it is amenable for SEM analysis which facilitates a direct morphological analysis of the formed structure on the surface. Within this work, we have studied the interaction between Aβ fibrils and ApoE where we, in addition, demonstrate how the technique of immunogold labeling can be combined to illustrate the binding pattern. Using this approach, the kinetics of the probed ligands, as well as the washing of each step to remove low and unspecific binding can be monitored prior to the morphological analysis.

## Results

### Amyloid formation of Aβ_1–40_ monitored by Thioflavin T (ThT) assay and TEM

**Fig. 1:**
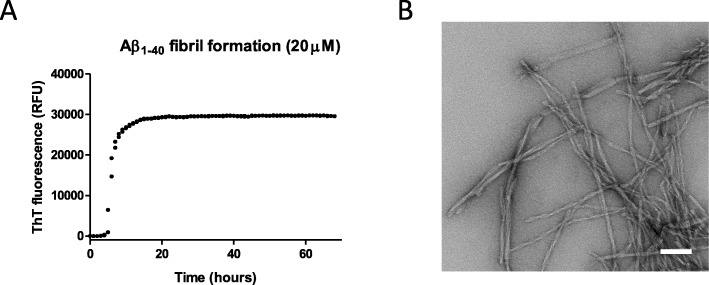
ThT and TEM analysis of fibril formation of Aβ_1–40_. **a** 20 μM monomeric peptide solution of Aβ_1–40_ was incubated in the presence of 40 μM of the amyloid reporting probe ThT. A steady state of the plateau indicates that monomeric fraction has been consumed and converted to the fibrillar form. **b** Negative uranyl acetate staining of the Aβ_1–40_ fibrils analyzed by TEM. Scale bar is 100 nm

Aβ_1–40_ readily forms fibrils under stagnant solutions in PBS and the relative proportion of fibrillar material can be monitored by using the amyloid-specific probe ThT. Figure 1a illustrates the kinetics of conversion of monomeric Aβ_1–40_ to a fibrillar form, where the initial lag-phase is followed by a logarithmic phase of fibril assembly, and subsequently reaches a plateau, where most of the monomeric fraction is converted to mature fibrils. Figure 1b illustrates a representative negative stain TEM image of the fibrils formed at the end of ThT kinetics wherein overall fibrillar morphology is developed.

### Surface Plasmon resonance

**Fig. 2:**
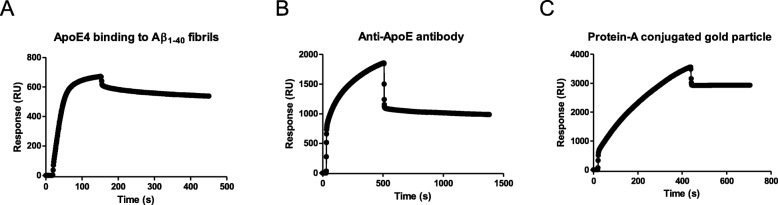
SPR analysis facilitates sequential probing of ApoE, anti-ApoE antibody and protein-A conjugated gold-particle. SPR analysis of **a** ApoE4 binding to immobilized Aβ_1–40_ fibrils, **b** ApoE4 bound Aβ_1–40_ fibrils probed with anti-ApoE antibodies, and **c** Protein-A gold beads binding to the fibril-ApoE-antibody complex. The analysis was performed in degassed PBS at pH 7.4

To study the binding of ApoE to Aβ_1–40_ fibrils using both SPR and SEM a scheme of analysis to include all controls was employed. In total 2000 response units (RU) of Aβ_1–40_ fibrils were immobilized and then probed with ApoE until saturation binding had been acquired implicating that most of the accessible sites have been occupied (Fig. 2a). The K_D_ of the binding between fibrils and recombinant human ApoE4 is strong and was calculated here to be around 5 nM, which is in overall good agreement with previous investigations [27, 28]. Bound ApoE4 was then probed through the addition of an anti-ApoE antibody, Fig. 2b. The removal of an excess of antibodies was effectively monitored using the SPR trace. As a last step 15 nm gold-beads conjugated to Protein-A was added and the binding monitored in analogy to previous steps, Fig. 2c.

### SEM-analysis of the CM5-chip surface

**Fig. 3:**
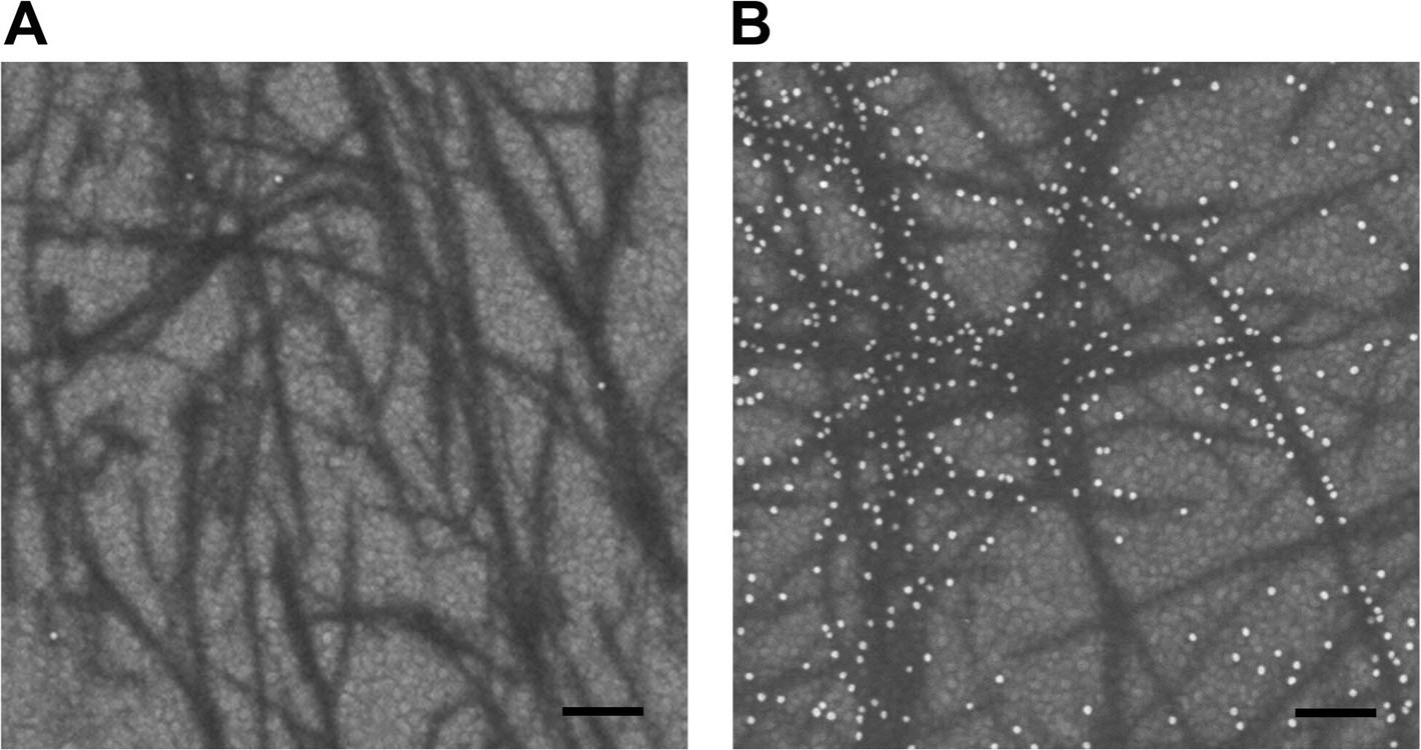
SEM analysis of the SPR-chip surface. **a** Control sample in absence of added ApoE to probe for non-specific binding where the immobilized fibrils on the SPR-chip have been sequentially probed with anti-ApoE antibodies and protein-A conjugated 15 nm gold-beads. **b** Complete setup where fibrils bound to the SPR-chip have been sequentially probed with ApoE4, anti-ApoE antibodies and protein-A conjugated 15 nm gold-beads. Scale bar is 100 nm

Due to the conducting substrate (gold-surface of the CM5-chip) it is possible to make direct analysis by SEM. Ultra- small (diameters < 10 nm), yet non-conducting materials such as the amyloid fibrils can be distinguished from the background by the inelastic electron scattering when the electron beam hits the sample surface at low accelerating voltage. The low-energy secondary electrons, detected by the in-lens detector, provide high-resolution, surface-sensitive information, as well as compositional contrast, displaying darker shades of gray for the organic matter (fibrils) and brighter gray level for the heavier elements (gold beads). The gold-beads indirectly probing bound ApoE could be readily identified due to their strong electron scattering properties and therefore easily discriminated from the background of both the gold-surface of the CM5-chip and the immobilized fibrillar sample. Figure 3a indicates a control surface area where all components of the described system, apart from ApoE, has been added.

The results revealed a fibrillar morphology in accordance with the TEM analysis of the corresponding sample and in essence no background binding is anti-ApoE antibodies and the subsequently added protein-A coated gold-beads was observed. Figure 3b shows the morphology of the sample after all components have been included. The results show how ApoE laterally decorates the amyloid fibrils, which is in accordance with previous findings using traditional immunogold techniques in combination with TEM [29, 30]. An apparent discrepancy can here be observed between different fibrils. While some fibrils are highly covered by the gold-beads others essentially completely lack any binding of ApoE, thus showing the absence of gold-beads. We observe the phenomenon of ApoE selectivity binding to certain fibrillar subtypes. However, due to the relatively low imaging resolution of SEM (compared to TEM) it is at this point of writing difficult to conclude if this is a technical artefact or if the observed results is based on a selectivity of ApoE for different types of fibrillar morphology.

### Discussion

Gold-particles have been used within the field of electron microscopy for a very long time due to their remarkable electron density and are today available in different sizes and conjugated to different linker molecules [31, 32]. In the present study we described an alternative immunogold labeling approach by using a combination of SPR and SEM to achieve both quantitative and morphological evaluation of the sample.

A limiting factor regarding the traditional use of immunogold techniques is that the sample is not strongly immobilized onto the surface. This feature frequently hampers the removal of unspecific binding after probing with e.g. antibodies or ligands and it is as a consequence difficult to acquire a good signal to noise ratio since an appropriate washing is difficult to perform and monitor. Sample preparation for TEM is also frequently associated with negative staining using a water solution of uranyl-acetate having pH between 4 and 5 which also may compromise the binding and may cause dissociation of assembled protein complexes.

Present work is focused on the interaction between the amyloid form of Aβ and its well-known ligand ApoE where in particular the ApoE ε4 allele is associated with a significantly increased risk of developing the disease [21, 33]. Through the current approach the fibrillar forms of the Aβ peptide are covalently attached to the activated SPR surface of a CM5-chip. After immobilization the sample is washed by a continuous flow of PBS to acquire a steady baseline and a sample amenable for the probing with ligands according to standard procedures. Detection of bound ApoE was further performed by an anti-ApoE antibody followed by protein-A conjugated to 15 nm gold beads. Apart from the possibility to determine the binding affinities and kinetics of the binding the technique enables monitoring of all washing steps where e.g. low affinity interactions effectively can be removed.

The procedure in more general terms exposes a strategy that may be employed also in other contexts and serves as a versatile tool whenever the ultra-structural morphology is of interest in combination with binding kinetics.

Interestingly, we observed an unexpected discrepancy where ApoE frequently did not bind uniformly to all fibrils within a sample. While certain fibrils were highly covered with ApoE, others in essence completely lacked bound ApoE. While this may be a technical artefact an alternative option is that ApoE may have a selective affinity for certain fibrillar morphologies. Although a single fiber follows a very high structural order, similar to the formation of a crystal, fibrils may differ from each other and Aβ samples frequently expose heterogeneous fibril morphology [1–3, 34]. This can be easily observed by TEM and is also seen within the fibrillar samples used here (shown in Fig. 1b) where at least four different types of fibril morphologies can be identified. The discrepancy in binding (shown in the SEM images) may indicate that ApoE selectively attaches to certain fibrillar structures. While SEM is desirable for direct analysis of amyloid fibrils on SPR-chip, the strong background signals and non-featureless morphology of the gold-surface may prohibit detailed investigation of the fibrils. Future studies are therefore required to distinguish if this is a technical artefact or if ApoE may have a different selectivity for different fibrillar morphologies.

**Fig. 4:**
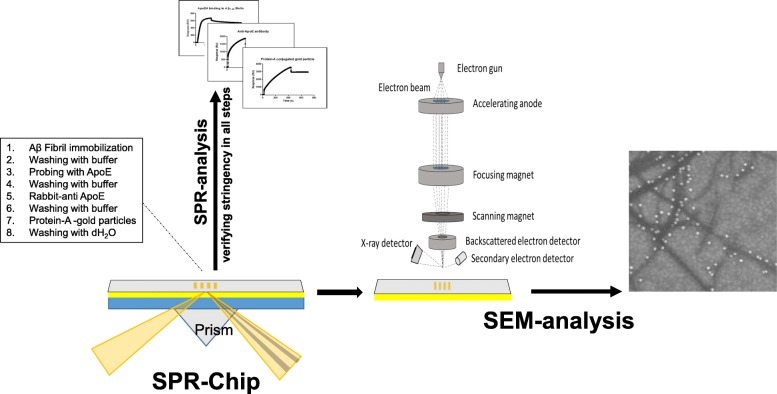
Schematic illustration of the setup using immunogold staining in combination with SPR and SEM

Taken together, we have demonstrated a combinatorial immunogold-labeling approach using SPR and SEM, which allows improving the stringency of the system of analysis. The setup facilitates both quantitative and morphological evaluation where importantly an efficient binding and washing can be monitored between each step in the sequential experimental approach defining the immunogold technique. The technique is in essence applicable to most setups using SPR where an ultrastructural morphology also is of interest. A schematic illustration of the setup is shown in Fig. 4.

## Methods

### Preparation of ApoE4 and Aβ_1–40_ monomers

Recombinant lyophilized ApoE4, as well as recombinant lyophilized Aβ_1–40_ peptides, were obtained from AlexoTech AB (Umeå, Sweden). Aβ_1–40_ was dissolved in 20 mM NaOH while both ApoE was dissolved in PBS. To remove potential aggregates both ApoE as well as Aβ_1–40_ were subjected to size-exclusion chromatography (Superdex 75 10/300, GE Life Science, Uppsala, Sweden) in degassed PBS at 4 °C prior to its use. Both Aβ_1–40_ as well as ApoE eluted as a single peak.

### Preparation of Aβ_1–40_ fibrils

In order to prepare Aβ_1–40_ fibrils, freshly gel-filtrated monomeric Aβ in PBS was distributed in a 96-well microtiter plate with dark walls and clear bottom (Cat. No.3881, Corning, USA) to a final concentration of 20 μM and incubated at 37 °C in the presence of 40 μM ThT. The aggregation process was monitored by recording the ThT signal using a fluorescence microplate reader (Tecan Infinite 200Pro, Männedorf, Switzerland) with excitation at 440 nm and emission at 480 nm according to standard procedures [35]. Fibrillar samples were considered ready when the binding of ThT reached a plateau.

### Negative staining transmission electron microscopy (TEM)

Prepared Aβ_1–40_ fibrils were sonicated for 60 s in a water bath and a total volume of 3.5 μl of fibrils was applied to 300 mesh formvar/carbon-coated, glow-discharged Ni-grids. After 1.5 min, the grid was washed in distilled water and consequently negatively stained for 15 s with 1.5% uranyl acetate (TAAB, Berks, England). Finally, fibrils were examined under a Talos L120 TEM (FEI) microscope (120 kV) equipped with a Ceta CMOS 4 k × 4 k pixel (FEI) camera supported with the FEI TIA (TEM imaging and analysis).

### Surface plasmon resonance

The interaction between Aβ fibrils and ApoE was monitored using a BIAcore 3000 biosensor (GE Healthcare, Uppsala, Sweden) equipped with a CM5 sensor chip (GE Healthcare). Prior to fibril immobilization the dextran matrix on the sensor chip surface was activated with a mixture of 1-ethyl-3- (3-dimethylaminopropyl) carbodiamide (EDC) and N-hydroxysuccinimide (NHS). Aβ fibrils were immobilized at a density of 2000 response unit (RU) using standard amino coupling reagents and then deactivated according to the manufacture instruction. All SPR experiments were performed in degassed PBS at 25 °C. Fibrils were immobilized at a flow rate of 5 μL/min, whereas Aβ_1–40_ monomeric peptide solution, ApoE4, Anti-ApoE (rabbit polyclonal, Cat# PA5–27088, ThermoFisher Scientific) and 15 nm protein-A gold beads (CMC Utrecht, Netherlands) were injected at a flow rate of 20 μL/min. All the steps were followed by a 5 min flow of buffer, and after the final step additionally washed by degassed distilled water for 5 min at a flow rate of 50 μL/min. Biosensor data were processed by BioLogic Software Scrubber2 and sensograms were made using GraphPad Prism 5.01.

### Scanning electron microscopy (SEM)

Prior to analysis the SPR chip was disassembled and mounted onto an aluminium stub using carbon adhesive tape and a copper tape is applied between the chip surface and metal stub for proper electrical grounding. The sample morphology was examined by field-emission scanning electron microscope (FESEM; Carl Zeiss Merlin GmbH) using an in-lens secondary electron (SE) detector at an accelerating voltage of 3 kV and probe current of 90 pA. The immobilized and Au-labelled fibrils can be directly visualized at low beam accelerating voltage without the application of a thin metal coating. Furthermore, no additional steps of sample processing are required e.g. fixation, dehydration and drying.

## Abbreviations

AD: Alzheimer’s disease
ApoE: Apolipoprotein E
Aβ: Amyloid β peptide
K_D_: Dissociation constant
RU: Response units
SEM: Scanning electron microscopy
SPR: Surface plasmon resonance
TEM: Transmission electron microscopy
ThT: Thioflavin T
TTR: Transthyretin
